# Examining volumetric gradients based on the frustum surface ratio in the brain in autism spectrum disorder

**DOI:** 10.1002/hbm.25270

**Published:** 2020-12-09

**Authors:** Caroline Mann, Tim Schäfer, Anke Bletsch, Maria Gudbrandsen, Eileen Daly, John Suckling, Edward T. Bullmore, Michael V. Lombardo, Meng‐Chuan Lai, Michael C. Craig, MRC AIMS Consortium, Simon Baron‐Cohen, Declan G.M. Murphy, Christine Ecker

**Affiliations:** ^1^ Department of Child and Adolescent Psychiatry, Psychosomatics, and Psychotherapy University Hospital, Goethe University Frankfurt am Main Germany; ^2^ Brain Imaging Center Goethe‐University Frankfurt am Main Germany; ^3^ Department of Forensic and Neurodevelopmental Sciences, and the Sackler Institute for Translational Neurodevelopmental Sciences Institute of Psychiatry, Psychology and Neuroscience, King's College London United Kingdom; ^4^ Brain Mapping Unit, Department of Psychiatry University of Cambridge Cambridge United Kingdom; ^5^ Autism Research Centre, Department of Psychiatry University of Cambridge Cambridge United Kingdom; ^6^ Laboratory for Autism and Neurodevelopmental Disorders, Center for Neuroscience and Cognitive Systems Istituto Italiano di Tecnologia Rovereto Italy; ^7^ Centre for Addiction and Mental Health and The Hospital for Sick Children, Department of Psychiatry University of Toronto Toronto Ontario Canada; ^8^ Department of Psychiatry National Taiwan University Hospital and College of Medicine Taipei Taiwan; ^9^ National Autism Unit Bethlem Royal Hospital London United Kingdom

**Keywords:** autism spectrum disorder, brain anatomy, computational neuroimaging, cortical folding, grey matter volume, gyrification

## Abstract

Autism spectrum disorder (ASD) is a complex neurodevelopmental disorder that is accompanied by neurodevelopmental differences in regional cortical volume (CV), and a potential layer‐specific pathology. Conventional measures of CV, however, do not indicate how volume is distributed across cortical layers. In a sample of 92 typically developing (TD) controls and 92 adult individuals with ASD (aged 18–52 years), we examined volumetric gradients by quantifying the degree to which CV is weighted from the pial to the white surface of the brain. Overall, the spatial distribution of Frustum Surface Ratio (FSR) followed the gyral and sulcal pattern of the cortex and approximated a bimodal Gaussian distribution caused by a linear mixture of vertices on gyri and sulci. Measures of FSR were highly correlated with vertex‐wise estimates of mean curvature, sulcal depth, and pial surface area, although none of these features explained more than 76% variability in FSR on their own. Moreover, in ASD, we observed a pattern of predominant increases in the degree of FSR relative to TD controls, with an atypical neurodevelopmental trajectory. Our findings suggest a more outward‐weighted gradient of CV in ASD, which may indicate a larger contribution of supragranular layers to regional differences in CV.

## Graphical abstract

During human brain development, the grey matter volume (CV) expands extensively, leading to formation of the gyral‐sulcal pattern of the cortical surface. Given the folded architecture of the cortex, the distribution of CV at a given location on the surface of the brain is likely to differ, depending on whether the cortex is *outward‐folded* (as in gyri) or *inward‐folded* (as in sulci). We have designed a novel imaging feature (FSR) to capture volumetric gradients from the outer to the inner surface of the brain, and to quantify the distortion of CV across cortical layers. This may be of relevance to neurodevelopmental disorders associated with atypical cortical expansion and lamination, as has been suggested for Autism Spectrum Disorder.

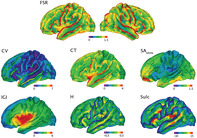

## 1 INTRODUCTION

The neural architecture of the cerebral cortex is complex and subject to dynamic changes throughout the human lifespan. During postnatal development, the cortex undergoes a period of extensive growth, which is characterized by a rapid expansion in surface area (SA), and an increase in cortical thickness (CT), leading to a commensurate increase in cortical grey matter volume (CV) (Giedd et al., 1999). While different mechanisms have been proposed to drive the increase in brain volume (Lewitus, Kelava, & Huttner, 2013), evidence suggests that grey matter development is not uniform across the cortical mantle, but differs across brain regions and cortical layers. For example, it has been noted that the upper and lower layers of the cortex expand differentially as a consequence of the tangential dispersion of radially migrating neurons (Reillo, De Juan Romero, García‐Cabezas, & Borrell, 2011). This generates a compressive force in the outer layer relative to the inner zone that has been suggested to cause folding of the cortex, and the formation of gyri and sulci (Kriegstein, Noctor, & Martinez‐Cerdeno, 2006; Richman, Stewart, Hutchinson, & Caviness, 1975). Differential expansion between the outer and inner layers affects measures of CT, which tends to be thicker in gyri than in sulci (Llinares‐Benadero & Borrell, 2019). The thickness of the cortex also varies across its six‐layer structure with supragranular layers (i.e., layer 1–3) being thicker in sulci than in gyri (Hilgetag & Barbas, 2006; Wagstyl et al., 2016). Given the folded architecture of the cortex and variability in CT, the distribution of cortical volume at a given location on the surface of the brain is therefore not uniform across cortical layers but seems to differ depending on whether the cortex is outward‐folded (e.g., in case of a gyrus) or inward‐folded (e.g., in case of a sulcus).

Yet, traditional volumetric neuroimaging studies estimate grey matter volume as the product of SA and CT at each cerebral vertex, which is also known as the *product method*. As noted by Winkler and colleagues, this approach introduces a considerable bias as it underestimates grey matter volume in cortical gyri where the outer surface area is larger than the inner, and overestimates grey matter volume in cortical sulci where the inner area is larger than the outer, hence overestimating total grey matter volume across hemispheres (Winkler et al., 2018). The product method therefore only serves as a rough approximation of the true grey matter volume at a given location on the cortical surface. In order to overcome this limitation, Winkler and colleagues (Winkler et al., 2018) proposed a more advanced way of estimating local CV by using the so‐called *analytic method*, where volume is not simply estimated as the product of SA and CT. Instead, each face (i.e., triangle) on the white matter surface and its matching face on the pial (i.e., outer) surface are used to define an oblique truncated pyramid, the volume of which is computed analytically without introducing additional error (Winkler et al., 2018). To date, this approach has become the default for surface‐based analyses of CV as implemented in FreeSurfer (http://surfer.nmr.mgh.harvard.edu/). While the analytic method has become state‐of‐the‐art for quantifying vertex‐wise estimates of grey matter volume, it does not contain any information on the exact position of a vertex on the folded surface of the brain. Based on the analytic method alone, it is therefore not possible to determine if a vertex is located on a gyral crown with an *outward‐weighting* of grey matter volume towards the pial surface, or on a sulcal fundus with an *inward‐weighting* of cortical volume towards the white matter surface (Waehnert et al., 2014).

In the present study, we utilized a surface‐based neuroanatomical feature designed to capture volumetric gradients from the outer to the inner surface of the brain, and thus to quantify the distortion of grey matter volume across cortical layers. We refer to this feature as *Frustum Surface Ratio* (FSR), which describes the degree to which the cortical volume at a given vertex is ‘skewed’ or ‘weighted’ towards the outer (i.e., pial) or inner (i.e., white matter) surface. As such, FSR describes the ratio of the inner and outer surfaces of a truncated pyramidal frustum. Our aim was (a) to characterize the normative spatially distributed pattern of variability in FSR across the cortical surface based on typically developing controls to assess the biological plausibility and interpretability of the measure, (b) to establish the relationship between variability in FSR and other morphometric features to identify the degree to which FSR measures unique and shared aspects of the cortical architecture, and (c) to utilize this feature to explore neuroanatomical differences in the brain of individuals with autism spectrum disorder (ASD) relative to typically developing (TD) controls. We chose to examine the brain in ASD in particular as genetic investigations suggest that ASD‐related genes are not expressed equally across cortical layers, but may predominantly affect either the superficial (Parikshak et al., 2013) or the inner layers (Willsey et al., 2013). Such layer‐dependent expression of ASD‐associated genes might therefore have a differential impact on the development of outer and inner layers, which cannot be quantified by approaches that examine either the outer or inner surface of the brain in isolation. As the degree of FSR depends on the relationship between outer and inner surfaces, it may be sensitive to geometric distortions driven by a layer‐specific neurodevelopment. Measures of FSR might be particularly well suited to characterize the complex neural architecture of the brain in ASD that has previously been shown to be atypical based on conventional measures of neuroanatomy (Amaral, Schumann, & Nordahl, 2008; Ecker, 2017; Stanfield et al., 2007).

## 2 METHODS

### 2.1 Participants

A total sample of 184 adult individuals was recruited and assessed at the Institute of Psychiatry, Psychology and Neuroscience (IoPPN), London (*n* = 89), and the Autism Research Centre, Cambridge (*n* = 95). The sample consisted of 92 typically developing (TD) controls (18–52 years of age, 51 males and 41 females), and 92 individuals with a confirmed diagnosis of ASD (18–48 years of age, 53 males and 39 females). Participants were matched on age, sex, and full‐scale intelligence quotient (fsIQ). All participants gave informed written consent in accordance with the ethics approval by the National Research Ethics Committee, Suffolk, England. Detailed information on diagnostic criteria as well as inclusion/exclusion criteria are presented in the supplement (see Supplementary Information S1).

### 2.2 MRI data acquisition

Scanning was performed at the IoPPN, London, and Addenbrooke's Hospital, Cambridge, using a 3 T GE Signa System (General Electric). A specialized acquisition protocol using quantitative T1‐mapping was used to ensure standardization of structural magnetic resonance imaging (MRI) scans across scanner platforms. This protocol has previously been validated and extensively described elsewhere (Deoni et al., 2008; Ecker et al., 2012), resulting in high‐resolution structural T1‐weighted inversion‐recovery images, with 1 × 1 × 1 mm resolution, a 256 × 256 × 176 matrix, TR = 1800 ms, TI = 850 ms, FA = 20°, and FOV = 25.6 cm. These images were subsequently used for surface reconstruction.

### 2.3 Image processing

Image processing and cortical reconstruction was performed using FreeSurfer v6.0.0 software (http://surfer.nmr.mgh.harvard.edu/). These well‐validated and fully automated procedures have been described in previous studies (Dale, Fischl, & Sereno, 1999; Fischl & Dale, 2000; Fischl, Sereno, & Dale, 1999; Jovicich et al., 2006; Ségonne et al., 2004). In brief, the processing pipeline includes intensity normalization, skull stripping (Ségonne et al., 2004), removal of extra‐cerebral tissue, volumetric labelling, and white matter segmentation using a connected components algorithm. Then, a triangular tessellated surface is generated for each white‐matter volume by fitting a deformable template, resulting in a cortical mesh for the white matter (i.e., inner) and pial (i.e., outer) surfaces. The resulting surface models were visually inspected for reconstruction errors and the quality of each scan was rated. Manual edits were performed by making changes to the pial (i.e., grey matter) outline, to the white matter outline, or both. Following manual editing, images were re‐preprocessed and re‐assessed for reconstruction errors. A summary of the quality assessments in terms of in‐ and excluded scans is provided in the supplementary material (see Supplementary Information S2).

All morphometric features were initially computed within the standard FreeSurfer pipeline based on the individuals' native surfaces (i.e., in native space). At each cerebral vertex, CT was computed as the average of the distance between a white surface vertex and the closest vertex on the pial surface (Fischl & Dale, 2000). Vertex‐wise estimates of SA were computed using the analytic approach outlined by Winkler et al. (Winkler et al., 2018), which quantifies SA as one third of the area of faces (i.e., triangles) incident to a vertex, as each face on the mesh has three vertices. Local gyrification was computed as described by Schaer et al. (Schaer et al., 2008). As implemented in FreeSurfer, the local gyrification index (*l*GI) is computed as the ratio between the area of the pial (grey matter) surface and the area of a constructed smooth ‘hull’ surface around the cortex. Thus, the *l*GI reflects the amount of cortex buried within the sulcal folds in the surrounding area (Schaer et al., 2008). Sulcal depth measures the height or depth of a point above or below an average surface and describes the amplitude of folding (Fischl, Sereno, & Dale, 1999). Maps of mean curvature (*H*) were computed as the average of the principle curvatures *k*_*1*_ and *k*_*2*_, and reflect the extent to which the cortex is curved at a certain vertex (Pienaar, Fischl, Caviness, Makris, & Grant, 2008).

### 2.4 Computation of vertex‐wise estimates of FSR

To capture the volumetric gradient from the outer to the inner surface of the brain, we estimated the degree of FSR at each vertex as the ratio between the expected volume (V_E_) and the actual volume (V_A_) at each vertex, or

$$\mathrm{FSR}=\frac{V_{E}}{V_{A}},$$

where V_E_ denotes the estimated cortical volume at a vertex computed as the product of vertex‐wise estimates of the outer surface area and cortical thickness (V_E_ = SA_pial_ * CT), and V_A_ is the actual volume at a vertex derived as proposed by Winkler and colleagues (Winkler et al., 2018). However, while we have used FreeSurfer to obtain surfaces for the computation of SA and CT, we did not use the FreeSurfer method to obtain cortical volume V_A_. Instead, we estimated the actual (analytical) volume V_A_ at each vertex as the volume of a truncated pyramid or prism based on the conventional (i.e., geometric) formula to calculate the volume of a pyramidal frustum:

$$V_{A}=\frac{1}{3}\mathrm{CT}\left({\mathrm{SA}}_{\text{pial}}+{\mathrm{SA}}_{\text{white}}+\sqrt{{\mathrm{SA}}_{\text{pial}}\;x\;{\mathrm{SA}}_{\text{white}}}\right),$$

where CT equals the height (i.e., the cortical thickness) at each vertex, SA_white_ is the surface area of the white matter surface, and SA_pial_ is the surface area of the corresponding vertex on the pial surface (i.e., outer grey matter surface). As such, V_A_ is measured as the volume of a pyramid truncated (i.e., cut) by a plane parallel to its base. In contrast to our geometric computation of V_A_, FreeSurfer computes vertex‐wise estimates of CV that are analytically based on the exact vertex positions (i.e., the volume is split into three tetrahedrons; Winkler et al. (2018)). FreeSurfer also takes into account that the bottom and top faces of the pyramid may not be aligned in parallel, which is neglected in our geometric approach. However, both approaches for the computation of V_A_ result in nearly equivalent values (*r* = .98, *p* < .001).

Besides, rather than simply computing the ratio of SA_pial_ to SA_white_ ($\text{Rati}o_{\mathrm{pw}}=\frac{SA_{\text{pial}}}{SA_{\text{white}}}$), a non‐linear term in the denominator was chosen to ensure that FSR values are normally distributed across the cortical surface (see Figure S3 for further details). FSR represents a ratio measure in units of proportions that can take values smaller or larger than 1, depending on whether a vertex is located on a sulcus or gyrus. Hence, FSR < 1 indicates that the volume is *inward‐weighted*, for example, in case of a sulcus, and FSR > 1 when the volume is *outward‐weighted*, as would be expected in case of a gyrus (see Figure S4).

< 1: *inward‐weighted* volume (i.e., sulcus)

FSR_(i)_

> 1: *outward‐weighted* volume (i.e., gyrus).

### 2.5 Statistical analysis

#### 2.5.1 Characterization of FSR in typically developing controls

We initially explored the spatial distribution of FSR within our subsample of typically developing controls (*n* = 92) to characterize the neurotypical distribution of our feature across the cortical surface, and to establish the biological plausibility of the feature. Here, we examined the distribution of FSR across the cortex using descriptive parameters such as the minimum (min), maximum (max), median (med), interquartile range (IQR), mean (M), and standard deviation (SD).

Next, we assessed the relationship between FSR and other morphometric features derived by FreeSurfer. For visualisation purposes, and to compare the spatial distribution of FSR values with other morphometric features, vertex‐wise FSR values were initially mapped to standard space. The same was done for CV, CT, SA_white_, SA_pial_, *l*GI, mean curvature *H* and sulcal depth. Across vertices on the fsaverage surface, the distribution of FSR was examined based on its mean value averaged across all TD. The statistical relationship between features was assessed using Spearman's Rank Correlation Coefficients (*ρ*) across vertices (and subjects) in native space. We examined Spearman's Rank Correlation Coefficients as not all morphometric features are normally distributed. For each feature, the vertex‐wise values of all 92 typically developing controls were concatenated vertically into a single column vector. The average vertex count per subject was ~290.000, so horizontally combining the vectors for all features resulted in a two‐dimensional matrix of size ~27,000,000 (datapoints) × 8 (features). Spearman's *ρ* was then computed between each pair of surface descriptors. Corrections for multiple comparisons were performed using the Bonferroni method (Bonferroni, 1963). In addition to these correlations, which combine data across subjects and vertices and thus allowed us to pool both inter‐ and intraindividual data, correlations where further examined across subjects and across vertices separately. A more detailed description of the methodological approach for these analyses can be found in the supplementary material (see Figure S5).

Following the initial computation in native space, we also examined the effect of registering FSR measures to the common space surface template (i.e., the FreeSurfer fsaverage surface). Furthermore, we examined the effect of spatial smoothing on the distribution of the parameter. To this end, the native space data of each subject was compared to the respective distribution on the high‐resolution FreeSurfer common standard space template with ~330,000 vertices across hemispheres. Common space registration is crucial for the comparison between groups and for applying cortical parcellation brain atlases (Fischl, Sereno, Tootell, & Dale, 1999). In order to identify the effects of spatial smoothing, the mean distributions of FSR measures were generated with several full‐width at half‐maximum (FWHM) surface‐based Gaussian kernels: 0 (no smoothing), 5, 10, and 15 mm. The distributions were then compared with the native space data and across smoothing kernels by computing the mean percentage of overlap between distributions across participants.

To examine the degree of normality of FSR at each vertex, we computed probability maps illustrating the likelihood of the data being normally distributed using Shapiro–Wilk tests across all typically developing controls. Shapiro–Wilk's *p* was then plotted at each cortical vertex for FSR data smoothed at FWHM 0, 10, and 20 mm. To compare FSR to other cortical descriptors, these probability maps where also produced for measures of *H*, SA_white_, CT, and CV, and are displayed in the supplementary materials.

#### 2.5.2 Between‐group analysis

Vertex‐wise statistical analyses were conducted using the SurfStat toolbox (https://www.math.mcgill.ca/keith/surfstat/) for Matlab (R2017b; MathWorks). We examined differences in FSR between ASD and TD, as well as age‐by‐group interactions by applying a general linear model (GLM) at each vertex i for subject j, with (a) group as categorical fixed‐effect factor, (b) an age‐by‐group interaction term, and (c) sex, site, fsIQ, and age as covariates, so that

$$Y_{\mathrm{ij}}=\beta_{0}+\beta_{1}{\text{Group}}_{j}+\beta_{2}\mathrm{Age}*{\text{Group}}_{j}+\beta_{3}{\mathrm{Sex}}_{j}+\beta_{4}{\text{Site}}_{j}+\beta_{5}{\mathrm{IQ}}_{j}+\beta_{6}{\mathrm{Age}}_{j}+\varepsilon_{i}$$

where ε_i_ denotes the residual error. Corrections for multiple comparisons across the whole brain were performed using random field theory (RFT)‐based cluster analysis for non‐isotropic images with a significance threshold of *p* < .05 (two‐tailed) (Worsley, Andermann, Koulis, MacDonald, & Evans, 1999). We also performed the between‐group analysis (a) with FSR smoothed at FWHM 5 mm, (b) with V_A_ being computed based on the FreeSurfer formula as suggested by Winkler et al. (2018), and (c) using mean Euler number as continuous covariate in the statistical model. Last, we compared our results of the FSR analysis with between‐group differences in traditional measures of CV. Here, we applied a smoothing filter of FWHM 10 mm in order to allow for comparability with other studies.

## 3 RESULTS

### 3.1 Neurotypical distribution of FSR measures across the cortex

**FIGURE 1:**
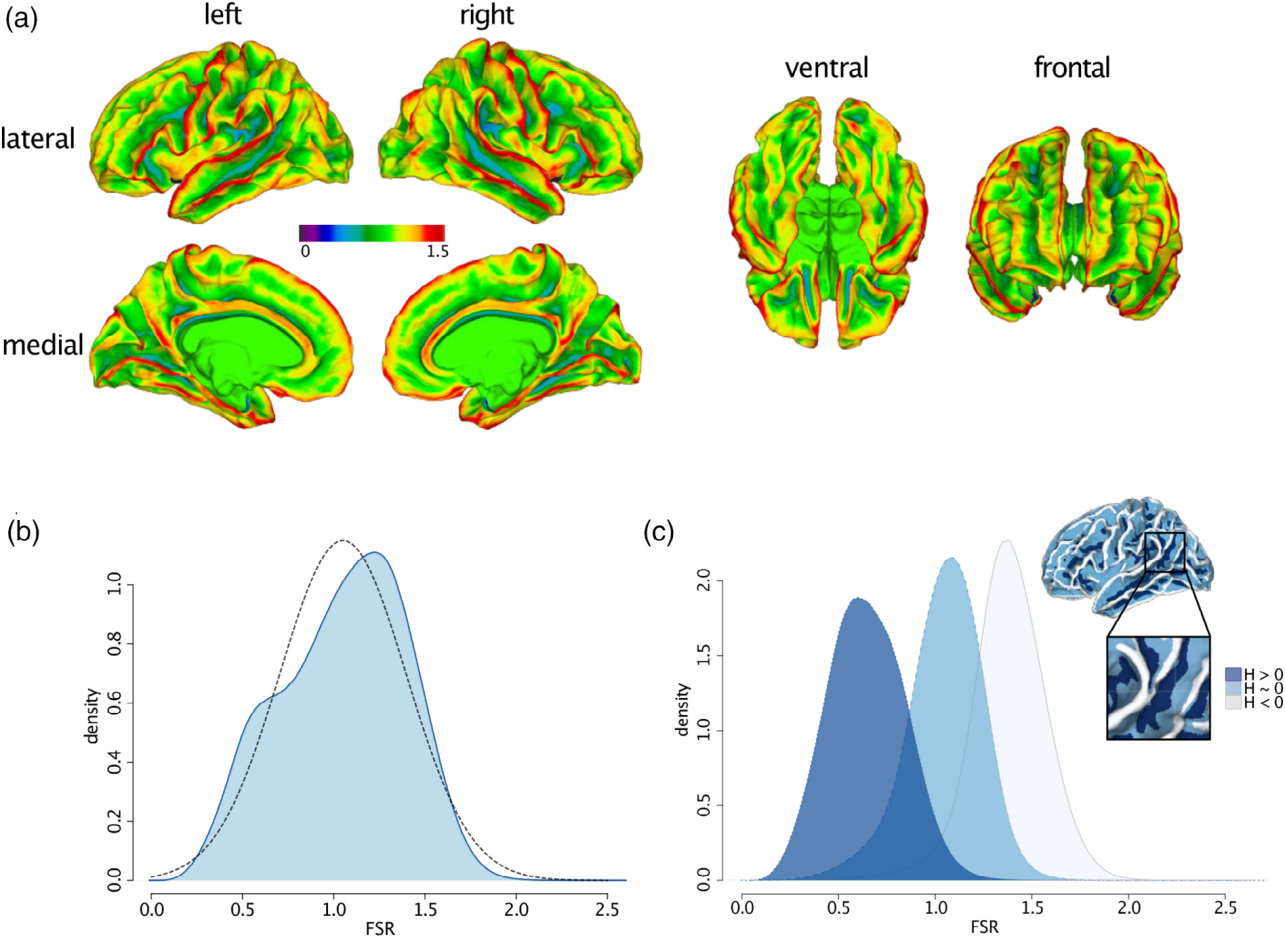
Spatial distribution of FSR in typically developing controls. (a) Spatially distributed pattern of FSR values averaged across TD mapped onto FreeSurfer common group template (fsaverage). Lower FSR values <1 (purple to cyan) indicate a more *inward‐weighted* cortical volume (CV) as is the case in a sulcal pit; medium FSR values indicate an even distribution of CV across cortical layers as is the case on a gyral wall (green); FSR values >1 indicate a more *outward‐weighted* CV as is the case on a gyral crown (yellow to red). (b) Distribution of mean FSR values (blue line) across the cortical surface approximating the Gaussian normal distribution (dotted line). (c) FSR density distribution across vertices located on sulci (dark blue), in close proximity of the gyral wall (light blue), and on a gyri (grey), subdivided based on the mean curvature (H) at a vertex. FSR, frustum surface ratio; H, mean curvature

The spatially distributed pattern of variability in FSR in TD is shown in Figure 1a. As expected, the degree of FSR follows the overall gyral and sulcal pattern of the cortical surface and tends to be higher in gyri than in sulci. Consequently, the distribution of FSR is a linear mixture of two Gaussian distributions ranging from a minimum value of 0.02 to a maximum value of 2.8 (first IQR = 0.79, med = 1.08, third IQR = 1.31, M = 1.05, SD = 0.35) (Figure 1b). To disentangle the bimodal distribution, and to the identify the influence of cortical folding on measures of FSR, we subdivided the cortex based on each vertex' mean curvature (*H*) into: (1) gyri (*H* ≤ −0.1), (2) gyral wall regions (−0.1 < *H* < 0.1), or (3) sulci (*H* ≥ 0.1) (for a similar procedure see Wagstyl et al., 2016). We then plotted the respective FSR distribution within each folding category. As can be seen in Figure 1c, the overall FSR distribution across the cortex is a linear mixture of vertex estimates in sulci (M = 0.65, SD = 0.20) or in close proximity to a gyral wall (M = 1.05, SD = 0.20), and of vertices located on gyri (M = 1.38, SD = 0.20). The overall spatial distribution of FSR therefore follows the overall pattern of cortical folding with a more *outward‐weighted* volume in gyri, and a more *inward‐weighted* volume in sulci.

**FIGURE 2:**
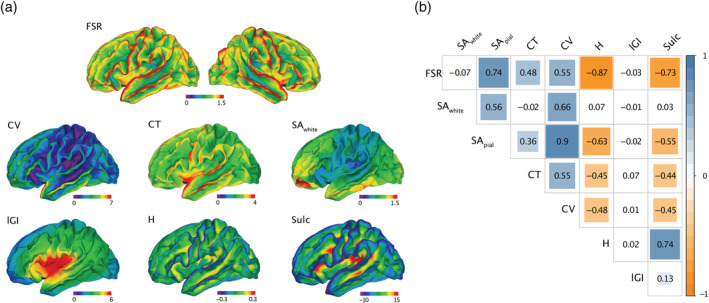
Relationship between FSR and well‐established morphometric descriptors. (a) Spatially distributed pattern of morphometric features across TD, displayed on the FreeSurfer default common group template (fsaverage). Lower values of the respective cortical feature are displayed in purple to cyan, higher values are displayed in yellow to red. (b) Spearman's rank correlation coefficients between morphometric features across vertices and subjects in native space. Negative correlations are displayed in orange, positive correlations are displayed in blue. FSR, frustum surface ratio; SA_white_, surface area of white surface; SA_pial_, surface area of pial surface; CT, cortical thickness; CV, cortical volume; H, mean curvature; lGI, local gyrification index; Sulc, sulcal depth

As shown in Figure 2a, the spatial distribution of FSR also closely follows the distribution of other high‐resolution geometric features that are typically computed either on the outer or inner brain surface. Consequently, measures of FSR were significantly correlated with several other morphometric features. More specifically, vertex‐wise estimates of FSR were significantly positively correlated with SA_pial_ (*ρ* = 0.74, *p* < .001), CT (*ρ* = 0.48, *p* < .001), and CV (*ρ* = 0.55, *p* < .001), and significantly negatively correlated with mean curvature *H* (*ρ* = −0.87, *p* < 0.001) and sulcal depth (*ρ* = −0.73, *p* < .001). Lower correlations, despite being significant due to the large number of vertices, were observed between FSR and *l*GI (*ρ* = −0.03, *p* < .001) and SA_white_ (*ρ* = −0.07, *p* < .001). Notably, although correlations were highly significant overall, no cortical feature explained more than 76% of variability in FSR_._ Thus, while the spatially distributed pattern of variability in FSR is similar to other cortical features, none of these descriptors was able to capture the neuroanatomical information uniquely provided by FSR. We also examined correlations between FSR and other morphometric features across subjects and vertices separately, which are shown in the Supplementary material (see Figure S5). These figures indicate that regardless of the examined dimension, we observed strong correlations between FSR and measures of curvature (*H*), which was also the cortical feature that explained the largest percentage of variability in FSR.

**FIGURE 3:**
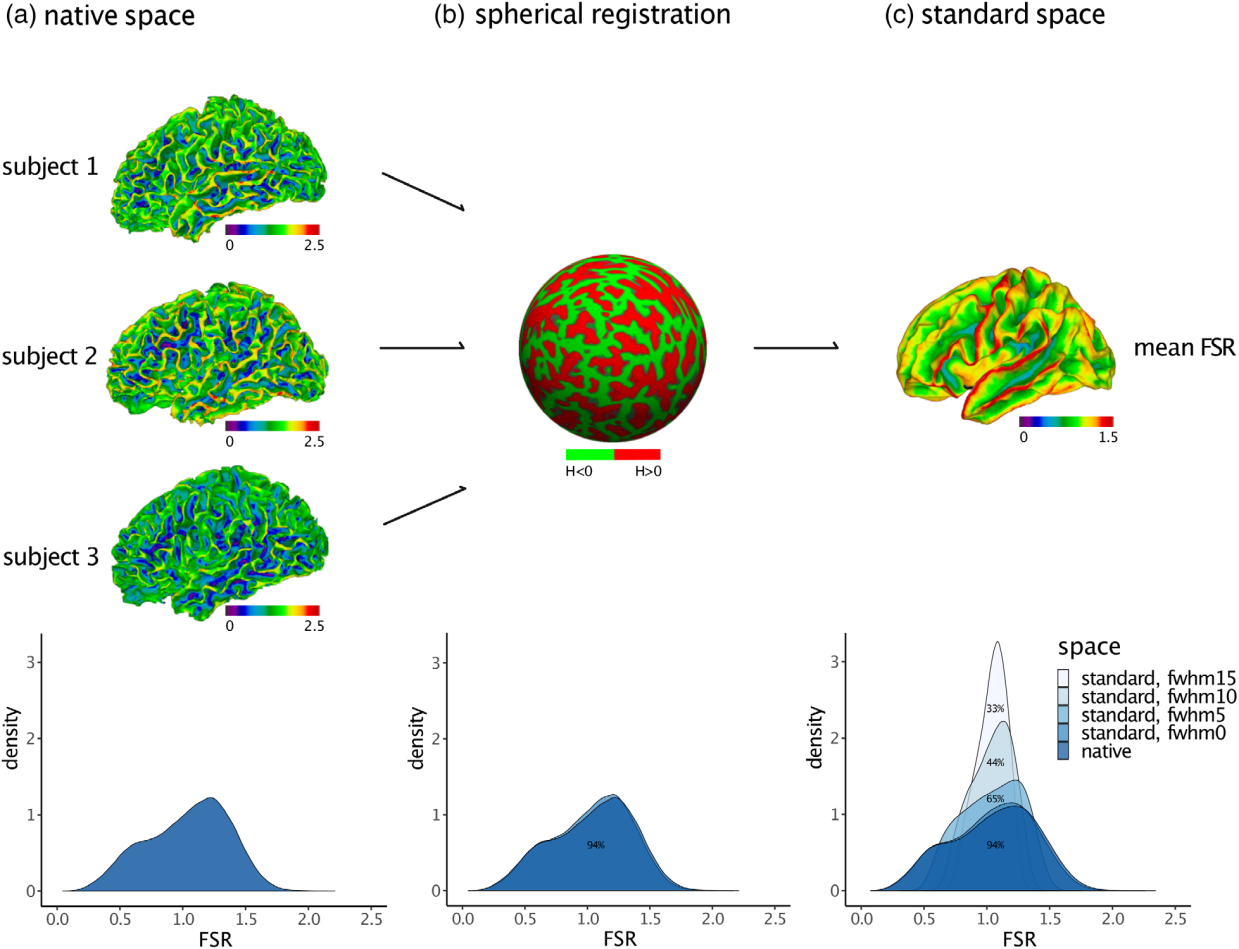
Common space registration and spatial smoothing. (a) FSR distribution for three representative TD in native space, and the respective native space density distribution of FSR across TD. (b) Distribution of FSR values in native space (dark blue) and following the registration into standard space (light blue) via spherical registration; numbers indicate the percentage of overlap between both distributions. (c) Distribution of mean FSR across TD in native and standard space, as well as after the application of different spatial smoothing kernels; numbers indicate the percentage overlap of distributions relative to the unsmoothed native space data. FSR, frustum surface ratio; H, mean curvature; fwhm, full‐width at half‐maximum

Last, we examined the effects of mapping FSR measures from native into standard space, as well as the effects of spatial smoothing. We observed that the distribution of FSR values did not change significantly as a result of spherical registration. Across TD, we observed a mean percentage overlap of 94% (*SD* = 0.06) between the FSR distribution in native space and in standard space (Figure 3b). Measures of FSR thus seem robust to common space registration. We did observe, however, a main effect of smoothing: larger smoothing kernels changed the distribution in terms of shape, symmetry, and mean value. As a result, the mean percentage of overlap between the FSR distribution in native space and the standard space distributions decreased from 94% at 0 mm to 33% at 15 mm smoothing (Figure 3c). Violin plots depicting an inverse association between percentage overlap and width of the smoothing kernel are presented in the supplementary material (see Figure S6). In order to keep distributional shifts induced by spatial smoothing as minimal as possible, we did not impose any smoothing for subsequent between‐group comparisons.

The results of the vertex‐wise analyses testing the degree of normality for measures of FSR and other features are presented in the supplementary material (see Figure S7). The probability of the data being normally distributed without smoothing (FWHM 0) across participants was relatively low for most features, and for SA_white_ and CV in particular. As expected however, the proportion of cortex areas with normally distributed data increased with higher smoothing settings and was highest at FWHM 20.

### 3.2 Vertex‐wise differences in FSR and CV between individuals with ASD and typically developing controls

For the clinical application, we examined differences in FSR in a sample of 92 participants with ASD compared to the 92 typically developing controls. Groups did not differ significantly in biological sex, mean cortical thickness, and total brain measures (*p* > .05; Table 1). We therefore did not covary for total brain measures in the vertex‐wise analysis of FSR.

**TABLE 1:** Participant demographics

	ASD	TD	Test statistic	
			χ^2^/*t*	*p*
n	92	92		
Sex (m/f)	53/39	51/41	0.02	.88
Site (London/Cambridge)	42 /50	47/45	0.35	.56
Age (years)	27 ± 7 (18–48)	28 ± 7 (18–52)	−1.62	.11
fsIQ	113 ± 13 (83–136)	116 ± 10 (93–137)	−1.69	.09
ADI‐R *social*	17 ± 5 (9–28)	–		
ADI‐R *communication*	13 ± 4 (7–24)	–		
ADI‐R *repetitive behaviour*	5 ± 2 (1–10)	–		
ADOS	8 ± 5 (0–18)	–		
Mean CT (mm)	2.67 ± 0.09 (2.46–2.89)	2.66 ± 0.08 (2.45–2.88)	0.51	.61
Total SA_white_ (m^2^)	0.19 ± 0.02 (0.15–0.26)	0.19 ± 0.02 (0.15–0.23)	0.87	.38
Total SA_pial_ (m^2^)	0.25 ± 0.02 (0.21–0.34)	0.24 ± 0.02 (0.19–0.29)	1.44	.15
Total CV (l)	0.78 ± 0.08 (0.61–1.06)	0.76 ± 0.07 (0.61–0.90)	1.12	.23
Total brain volume (l)	1.25 ± 0.14 (1.01–1.72)	1.24 ± 0.12 (0.95–1.48)	0.81	.42

*Note:* Data expressed as mean ± *SD* (range). There were no significant between‐group differences in age, full‐scale IQ, or any of the global brain measures at *p* < .05 (two‐tailed).

Abbreviations: ADI‐R, autism diagnostic interview–revised; ADOS, autism diagnostic observation schedule; CT, cortical thickness; CV, cortical volume; f, female; fsIQ, full‐scale intelligence quotient; l, liter; m, male; m, metre; mm, millimeter; *n*, number of participants per group; *p*, p‐value; SA_pial_, Surface Area of pial surface; SA_white_, Surface Area of white surface.

**FIGURE 4:**
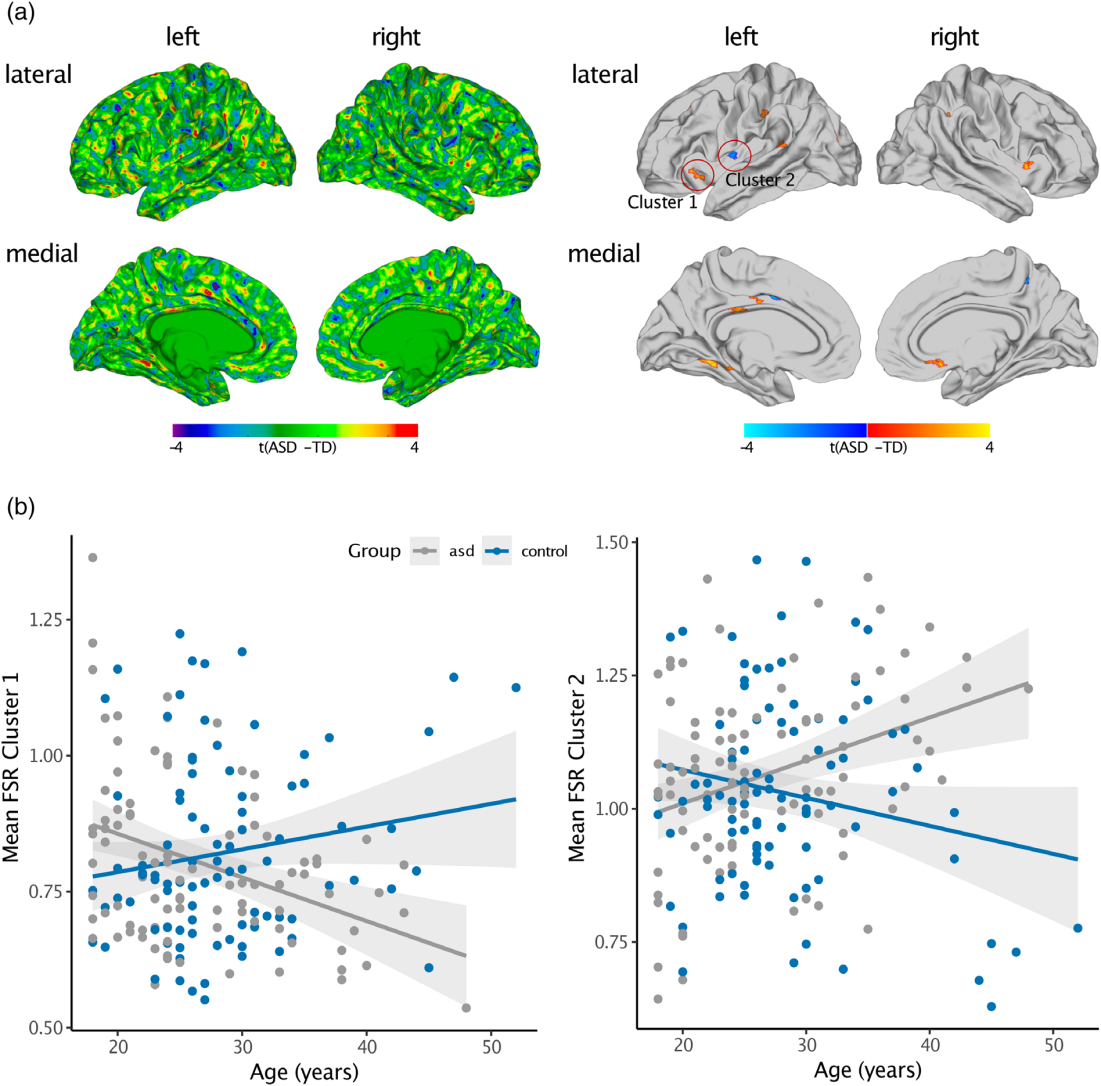
Between group differences and age‐by‐group interactions in FSR. (a) Significant differences in FSR between individuals with ASD and TD while controlling for the effect of age, age‐related interactions, sex, site, and fsIQ (i.e., main effect of group). *Left panel*: unthresholded *t*‐maps. *Right panel*: random field theory (RFT)‐based cluster corrected *t*–maps (*p* < .05, two‐tailed). Here, significant increases in FSR are displayed in red to yellow, significant decreases are displayed in blue to cyan. (b) Scatter plots depicting age‐related trajectories for individuals with ASD (grey) and TD (blue). *Left panel*: Age‐by‐group interactions in cluster located on the left orbitofrontal cortex. *Right panel*: Age‐by‐group interactions in cluster located on the left insula. ASD, autism spectrum disorder; TD, typically developing controls; *t*, *t*‐statistic; FSR, frustum surface ratio

Individuals with ASD significantly differed from TD controls in FSR in several regions across the cortex (Figure 4a and Table 2). Across the cortex, we observed predominantly increased FSR in ASD in the left middle frontal cortex (Brodmann area [BA] 46), the left lateral orbitofrontal cortex (BA 45), bilateral pre‐and postcentral gyri (BA 1–4), right superior and left inferior parietal cortex (BA 7), right insula (BA 13), left superior temporal lobe (BA 41), left parahippocampal cortex (BA 35), left fusiform gyrus (BA 37), right anterior cingulate cortex (ACC; BA 24), and left posterior cingulate cortex (PCC; BA 23). In these brain regions, ASD individuals showed a larger gradient in brain volume from the outer to the inner cortical surface, with a more *outward‐weighted* volume compared to TD controls. There were also a few brain regions where we observed decreased FSR in ASD, which included the left postcentral gyrus (BA 1–3), left precuneus (BA 7), left insula (BA 13), and left PCC (BA 23). In these brain regions, cortical volume was distributed more towards the inner cortical surface in ASD (i.e., *inward‐weighted*). When increasing the smoothing size to FWHM 5 mm, between‐group differences were reduced to one significant cluster in the inferior parietal cortex, where FSR was increased in ASD compared to typically developing controls (see Figure S8A). However, the pattern of between‐group differences did not change when computing V_A_ based on the FreeSurfer formula (see Figure S8B), or when including mean Euler number as continuous covariate (see Figure S8C). For the between‐group comparison of vertex‐wise measures of cortical volume (CV), see Figure S9. Notably, there was no spatial overlap between the pattern of differences in CV and FSR indicating FSR measures aspects of the cortical architecture that are not captured by vertex‐wise measures of CV alone.

**TABLE 2:** Cluster information on between‐group differences in FSR

Cluster	Regional labels	Side	BA	Vertices	*t*_*max*_	*p*	Talairach coordinates x y z
**Between‐group difference in FSR**							
1	Posterior cingulate cortex	L	23	129	3.46	1.36 × 10^−4^	−8 −25 28
2	Anterior cingulate cortex	R	24	93	4.15	1.01 × 10^−2^	7 14–11
3	Inferior parietal cortex	L	7	150	3.33	5.16 × 10^−4^	−37 −81 21
4	Parahippocampal gyrus	L	35	108	2.99	1.09 × 10^−3^	−25 −40 −6
5	Precentral gyrus	R	4	80	3.30	1.26 × 10^−3^	33–22 43
6	Lateral orbitofrontal cortex	L	45	105	3.26	2.52 × 10^−3^	−26 22–6
7	Superior parietal cortex	R	7	195	3.63	5.37 × 10^−3^	33–45 37
8	Supramarginal gyrus	L	40	182	4.34	5.90 × 10^−3^	−56 −30 36
9	Postcentral/supramarginal gyrus	L	1,2,3,40	128	3.14	7.62 × 10^−3^	−38 −28 35
10	Posterior cingulate cortex	L	23	113	3.48	7.96 × 10^−3^	−7 −15 36
11	Fusiform gyrus	L	37	122	3.94	1.58 × 10^−2^	−34 −48 −5
12	Insula	R	13	58	3.63	2.11 × 10^−2^	33 11–1
13	Superior temporal gyrus	L	41	136	3.19	2.34 × 10^−2^	−59 −41 16
14	Insula	L	13	80	3.71	3.26 × 10^−2^	−37 −5 17
15	Rostral middle frontal gyrus	L	46	157	3.47	3.29 × 10^−2^	−28 24 32
16	Postcentral gyrus	L	1,2,3	145	−1.65	7.84 × 10^−3^	−54 −10 14
17	Precuneus	L	7	112	−1.66	8.64 × 10^−3^	−16 −55 18
18	Insula	L	13	59	−1.67	1.74 × 10^−2^	−34 −5 9
19	Posterior cingulate cortex	L	23	108	−1.66	4.91 × 10^−2^	−5 1 35
20	Precuneus	R	7	111	−1.65	4.96 × 10^−2^	8–44 48

Abbreviations: BA, approximate Brodmann area(s); *FSR*, Frustum Surface Ratio; L, left; *p*, cluster‐corrected *p*‐value;R, right; *t*_max_, maximum *t*‐statistic within cluster; Vertices, number of vertices within the cluster.

#### 3.2.1 Effect of age on FSR

In many regions with significant between‐group differences in FSR we also observed significant age‐by‐group interactions. Linear age‐by‐group interactions were observed in the bilateral insulae (BA 13), precentral gyri (BA 1–3), PCC (BA 23), and inferior parietal cortices (BA 7), as well as in the left supramarginal gyrus (BA 40), left superior temporal gyrus (BA 41), and left middle frontal, superior frontal, and orbitofrontal regions (BA 6, 45, 46). Here, were observed a negative association between FSR and age in the ASD group, and a positive correlation in the TD group. Additionally, we identified linear age‐by‐group interactions in the bilateral insulae (BA 13), left PCC (BA 23), and left inferior parietal cortex (BA 7). In these regions, we observed a positive association between FSR and age in the ASD group and a negative correlation in the TD group (see Figure 4b for two exemplary scatterplots of age‐by‐group interactions). Thus, while we did observe a significant main effect of group, our findings suggest that between‐group differences in FSR are age dependent during adulthood.

## 4 DISCUSSION

In this study, we examined volumetric gradients from the outer to the inner cortical surface in TD, and in individuals with ASD, using a novel cortical feature that relates the expected CV based on the surface area of the cortical pial surface to the actual CV computed at each cerebral vertex. The resulting measure indicates the amount to which cortical volume is distorted from a regular (triangular) prism to a more cone‐ or pyramid‐shaped 3D object. Such distortions have already been described by Bok (1929) and have guided similar analytical frameworks to model anatomically meaningful locations of cortical laminae (Waehnert et al., 2014). In these studies, an *equivolume* model is assumed, in which the cortical volume is considered constant in neighboring segments of a given layer. Similarities thus exist between the equivolume approach and the mesh generation algorithm used in FreeSurfer, where the local vertex density on the surfaces positively correlates with their absolute curvature, that is, more vertices are placed in regions of high curvature. Therefore, the shape of the pyramidal frustum defining vertex‐wise CV adapts based on changes in local curvature. This degree of adaptation is also quantified by measures of FSR, which hence offers information that cannot adequately be captured by traditional measures of CV alone.

### 4.1 Characterizing FSR in native space

Based on our typically developing controls, we established that the degree of FSR follows the overall gyral and sulcal pattern of the cortical surface, where cortical volume is skewed towards the outer or inner surface depending on whether a vertex is located on a gyrus or sulcus. Moreover, measures of FSR follow an anterior–posterior gradient resembling the spatial gradients of volumetric features (i.e., CT and CV). The observed high correlations of FSR with other descriptors, and curvature measures in particular, may in part be explained by local effects of the mesh generation algorithm employed by FreeSurfer. In highly curved areas, vertices are placed closely together to satisfy smoothness constraints, which means that each vertex in a densely packed region has a relatively small SA. In gyri, where the curvature of the outer surface is smaller than the inner, smaller SAs are designated to the white matter surface relative to the pial surface, leading to larger FSR values. In sulci, where the curvature of the outer surface tends to be larger than the inner, smaller SAs are designated to the pial surface relative to the white matter surface, leading to lower FSR values. This may also explain the observed positive correlations of FSR with CT and SA_pial,_ but not SA_white_, which may be a consequence of the cortex being slightly thicker in gyri (where FSR values are higher) than in sulci (where FSR values are lower). In gyri, the curvature of the pial surface is comparably low (i.e., lower than the curvature of the white surface), and thus larger vertex distances, and hence face areas, are produced by the non‐isoform mesh generation algorithm used in FreeSurfer. This does not affect the white surface to the same degree, where the tips of the gyri show higher absolute curvature values compared to the pial surface. The surface area of the white matter surface is therefore smaller than the pial surface, which could also explain the high correlation with SA_pial_ but not SA_white_. However, unlike curvature measures which describe either the outer or inner cortical surface in isolation, the degree of FSR captures the relationship between both surfaces and reflects unique aspects of the cortical architecture that cannot be adequately described based on a single surface alone. FSR is therefore one of the few morphometric features that combines information across cortical layers. This is of particular relevance for the characterization of disorders with a layer‐specific pathophysiology and aberrant cortical lamination, as has been suggested for ASD (Varghese et al., 2018).

### 4.2 Common space registration and spatial smoothing of FSR

In order to examine interindividual variability or group differences, it is necessary to map cortical features from the individual's native space geometry of the brain to a common standard‐space template (Drury et al., 1996). In FreeSurfer, FSR values were mapped from native to standard geometry via spherical transformation, which preserves the quantities originally assigned to vertices on a local, regional, and global level (Dale et al., 1999; Fischl, Sereno, Tootell, et al., 1999; Winkler et al., 2012). As measures of FSR are a novel cortical feature with unknown spatial and distributional characteristics, we initially examined the amount of distortion introduced by mapping the data onto the standard high‐resolution surface coordinate system provided by FreeSurfer (fsaverage) (Fischl, Sereno, & Dale, 1999). In general, the amount of distortion introduced by the common space registration is positively correlated with the spatial frequency of the feature. Features with an extremely high spatial frequency in native space, such as vertex‐wise measures of Gaussian curvature (Ronan et al., 2012), cannot be transformed into standard space without incurring a significant distortion of the distributional characteristics of the feature across vertices. These features are thus unsuited for the vertex‐wise comparison across individuals. Features with an inherently low‐resolution and a smooth spatial distribution across the cortical surface, such as the local gyrification index (Schaer et al., 2008), remain relatively unaffected by common space registration. For FSR we observed a percentage overlap between the native and standard space distributions of 94% across all individuals. Additionally, common space registration did not significantly affect the shape of the distribution, which retained its bimodality caused by a linear mixture of values on gyri and sulci. Our results therefore suggest that measures of FSR can be reliably mapped to a standard geometry without incurring significant information loss and are thus well suited for vertex‐wise comparisons between groups.

However, while measures of FSR seem relatively insensitive to common space registration, their distribution across the cortex significantly depend on the amount of spatial smoothing applied to the data. More specifically, there was a reduction in the percentage overlap between the distribution of FSR in native space and in standard space by 61% when increasing the smoothing kernel from 0 to 15 mm FWHM. Even when applying a smoothing filter of 5 mm in standard space, the percentage overlap between the FSR distribution in standard space relative to the native space decreased by 29%. Thus, in order to preserve the distribution of FSR values across the cortical surface, a minimal amount of smoothing (i.e., FWHM 0 or 5 mm) should be applied when examining group comparisons. Inter‐individual differences in FSR are therefore expected to be small and localized rather than affecting large‐scale cortical regions. In a next analysis step, we investigated the potential of FSR to detect statistical effects on the group level in order to characterize novel aspects of the neuroanatomical underpinnings of ASD.

### 4.3 FSR in adults with ASD

ASD is a highly heterogeneous condition that has been linked to neurodevelopmental differences in brain anatomy (Ecker, Bookheimer, & Murphy, 2015). This includes volumetric differences (Courchesne, 2004; Hazlett et al., 2017; Hazlett, Poe, Gerig, Smith, & Piven, 2006; Lange et al., 2015) as well as geometric differences such as atypical cortical folding (Ecker et al., 2016; Schaer et al., 2013; Wallace et al., 2013; Yang et al., 2016). While little is currently known about the aetiological mechanisms mediating cortical abnormalities in ASD, genetic evidence suggests that ASD risk genes are enriched among gene modules coregulated during cortical layer formation. More specifically, ASD‐associated modules have been reported to be specifically expressed in superficial cortical layers (i.e., layers 2,3,4), and are strongly associated with markers of upper layer glutamatergic neurons in the adult cortex (Parikshak et al., 2013). In addition, a disorganization of the cortical laminar architecture has been shown in post‐mortem brain tissue of ASD individuals, with the clearest signs of abnormal expression observed in lower cortical layers in ASD (i.e., layer 4,5,6) (Stoner et al., 2014; Willsey et al., 2013). Thus, while genetic investigations remain in disagreement with regards to which layers are the most affected in ASD, it is likely that some layers of the cortex are more affected than others. Here we demonstrate that measures of FSR were significantly increased in adult individuals with ASD in many areas across the cortex including frontal regions, anterior and posterior cingulate cortices, the insula, as well as parietal and temporal regions. In these brain regions, many of which have previously been linked to the set of behavioral symptoms characteristic for ASD (Amaral et al., 2008; Ecker, 2017), an increase in FSR indicates that the distribution of cortical volume is more *outward‐weighted*, that is, there is a steeper volumetric gradient from the inner to the superficial cortical layers. In contrast, only a few regions displayed a significant decrease in FSR in ASD (i.e., a more *inward‐weighted* volumetric gradient), which implicates particularly the infragranular cortical layers in the pathophysiology of ASD. Notably, this hypothesis is also supported by studies examining preclinical (i.e., rodent) models of ASD, where experimentally induced structural differences in the upper neocortical layers of mice have been suggested to result in autism‐like behavioural abnormalities (Fang et al., 2014). In addition to the main effect of group, we also observed significant age‐by‐group interactions, indicating that between‐group differences in FSR are age‐dependent, with differences being most prominent at an age of 40 years and above. A steeper decrease of FSR values indicates how the distribution of cortical volume is changing shape from a higher degree of *outward‐weighting* towards *inward‐weighting*. Such changes may reflect an age‐related decrease in the ‘steepness’ of gyri, that is, both the convexity of the gyral ridges and the curvature may decrease across the lifespan—leading to a more ‘atrophic’ brain in ASD in the observed brain regions. The results of a steeper age‐related decline of FSR are also in line with most previous literature examining cortical development in adults with ASD, with similar developmental trajectories being reported for cortical volume (Lange et al., 2015), cortical thickness (Zielinski et al., 2014), and gyrification (Kohli et al., 2019). Measures of FSR might thus be utilized as an age‐sensitive marker of the pathophysiology of ASD in adults and may guide future genetic studies into the aetiological underpinnings of the condition.

### 4.4 Limitations and outlook

The results presented in this study should be interpreted in the light of several limitations. First, we employed a multi‐site study design to overcome single‐site recruitment limitations. However, anatomical measures derived by FreeSurfer have been shown to be highly reliable across field‐strength and scanner‐platforms when controlling for MRI hardware and data processing (Han et al., 2006). Additionally, we have applied specialized scanning sequences to ensure a standardized acquisition of MRI scans across sites. Furthermore, the same pre‐processing pipeline and quality assessments were applied to all surface reconstructions, and inter‐site effects were controlled for the statistical model. Second, we examined FSR in a sample of adults aged between 18 and 52 years without intellectual disability. Given the large heterogeneity associated with ASD, our results may therefore not generalise to other (i.e., younger) populations on the autism spectrum, or to individuals with intellectual disability. Third, the choice of smoothing filter and multiple comparison corrections are inherently challenging for features with high resolution, such as FSR. Based on our finding that measures of FSR are highly susceptible to spatial smoothing, we performed the between‐group analysis using the un‐smoothed data and applied a random‐field theory (RFT)‐based cluster threshold (Worsley et al., 1999). While cluster‐extent based thresholding‐approaches are widely used to control for false positives in mass‐univariate analysis, these identify statistically significant clusters on the basis of voxel contiguity and are thus sensitive to the smoothness of the data. When increasing the smoothing size to 5 mm, between‐group differences were ‘smoothed out’ and reduced to one significant cluster in the inferior parietal cortex, where FSR was increased in ASD compared to typically developing controls. Thus, smaller smoothing kernels are better placed to capture between‐group differences yet limit the ability to detect significant effects on the cluster level. Due to (a) the high spatial frequency of FSR, which did not allow us to apply a larger smoothing filter, and (b) the size of effects and their spatial distribution (i.e., small, isolated peaks), a cluster threshold approach may not be the best option. Future validation using alternative (i.e., permutation‐based) approaches is required to replicate our findings. Moreover, ASD is a highly heterogeneous condition and low effect sizes are typically observed in studies examining differences in neuroanatomy (e.g., reviewed in Ecker et al., 2012). Thus, while the development of our feature was motivated by genetic studies suggesting a differential expression of genes across layers in ASD, the neuroanatomical differences associated with ASD are inherently difficult to describe. Future studies in other psychiatric conditions with a known layer‐specific development, such as schizophrenia, might therefore be useful to validate our feature. Hence, the results of the between‐group comparison should be considered preliminary, and it will be important to investigate the robustness of our results using (a) larger samples including different ASD subgroups and/or other clinical cohorts, and (b) using alternative statistical approaches that do not rely on the spatial distribution of effect sizes across the cortex, and that are more sensitive to local effects in isolated brain regions. Last, while we employed stringent data quality assessments including manual editing of the FreeSurfer surfaces, our approach is not immune to smaller reconstruction errors. Future research is needed to replicate our findings in larger samples of ASD individuals and TD following the same quality controls applied in the current investigation. This will also be important with regards to the large phenotypic heterogeneity associated with ASD that might be parsed on the neuroanatomical level using several different neuroanatomical features, including measures of FSR.

## CONFLICT OF INTERESTS

None of the remaining authors have declared any conflict of interest or financial interests, which may arise from being named as an author on the manuscript.

## AUTHOR CONTRIBUTIONS

Christine Ecker, Meng‐Chuan Lai, John Suckling, Edward T. Bullmore, Michael V. Lombardo, Michael C. Craig, Simon Baron‐Cohen, and Declan G.M. Murphy have conceptualised and designed the study. Maria Gudbrandsen, Eileen Daly, and Meng‐Chuan Lai have collected the data. Caroline Mann and Tim Schäfer have analysed and interpreted the data, provided FSR measures, and have drafted the manuscript. Caroline Mann, Tim Schäfer, and Christine Ecker have performed statistical analyses. Caroline Mann has written and revised the final manuscript. All co‐authors have revised the manuscript and provided important intellectual content. Edward T. Bullmore, Michael C. Craig, Simon Baron‐Cohen, and Declan G.M. Murphy have obtained funding. Christine Ecker has supervised the study.

## Supporting information


**Appendix** **S1:** Supporting information

## Data Availability

Data and code is made available via request to the authors.
