# Supplementary material for: Examining volumetric gradients based on the frustum surface ratio in the brain in autism spectrum disorder
Source: Hum Brain Mapp. 2020 Dec 9;42(4):953–66. doi: 10.1002/hbm.25270 (PMC7856638; doi:10.1002/hbm.25270)
Supplement: Supplementary file 1 — Appendix S1: Supporting information [file HBM-42-953-s001.pdf]

S1. Additional information on participant demographics.

ASD diagnosis was based on ICD-10 research diagnostic criteria and confirmed using the Autism Diagnostic Interview - Revised (ADI-R; Lord et al. 1994). The ADI-R rather than the Autism Diagnostic Observation Schedule, Second Edition (ADOS-2; Lord et al. 2000) was employed as inclusion criteria to ensure that all participants with ASD met the criteria for childhood autism. ADI-Rs were completed for 88 individuals with ASD (53 males and 35 females). All individuals reached algorithm cut-offs for autism in all domains of the ADI-R (social interaction, communication, and repetitive behaviour), although failure to reach cut-off in one domain by one point (or two points in the repetitive behaviour domain) was permitted. We were unable to complete ADI-Rs for four females with ASD as their parents/caregivers were not available. However, all four individuals reached algorithm cutoffs for 'autism spectrum' or 'autism' on the ADOS diagnostic algorithm. In all other participants, the ADOS-2 and the Autism-Spectrum Quotient (AQ) were used to assess current symptoms but were not used as inclusion criterion. Exclusion criteria included contraindications to MRI, a history of major psychiatric disorder (e.g. psychosis), head injury, genetic disorders associated with autism (e.g. fragile-X syndrome, tuberous sclerosis), any other medical condition affecting brain function (e.g. epilepsy), or any participants taking antipsychotic medication, mood stabilizers or benzodiazepines. Overall intellectual ability was assessed using the Wechsler Abbreviated Scale of Intelligence (WASI; Wechsler 1999). All participants had a full-scale intelligence quotient (fsIQ) greater than 80.

S2. Information on Quality Assessments and Manual Edits.

For 266 structural T1-weighted scans, the surfaces models derived by FreeSurfer were visually inspected for reconstruction errors. Quality was assessed by three raters blind to diagnosis based on the following criteria: (1) accept as is (good quality, no visible reconstruction errors or artifacts), (2) prescribe manual edits (visible reconstruction errors in either pial or white matter surface, or both, that may be recovered by manual editing), or (3) exclude (gross anatomical abnormalities or severe acquisition or motion artifacts). Initially, 15 (i.e. 6% of the total sample) scans were excluded due to insufficient quality. Manual edits were performed on 78 scans by making changes to the pial (i.e. grey matter) outline, to the white matter outline, or both. Images were re-preprocessed and re-assessed for reconstruction errors, which led to exclusion of 28 additional scans that did not improve after manual editing. After the quality assessments, 39 participants were further excluded in order to obtain two groups matched on age, gender, and full-scale IQ. This procedure resulted in a final sample size of 184 participants.

S3. Additional information on the relationship of FSR, Ratio_{pw}, and mean curvature *H*.

The descriptor FSR is computed as the ratio of V_E (the product of the pial surface area and cortical thickness), and V_A (a non-linear term including both pial and white matter surface areas multiplied by cortical thickness). As such, it was designed to capture the ratio of outer to inner surfaces of a frustum. In contrast to computing a simple ratio of pial to white surface areas ($\text{Ratio}_{\text{pw}} = \frac{\text{SA}_{\text{pial}}}{\text{SA}_{\text{white}}}$), the inclusion of the non-linear term in the ratio's divisor was chosen to ensure the normal distribution of FSR. The distributions of both white matter and pial vertex-estimates of surface area are heavily skewed as a consequence of the non-uniform surface mesh generated by FreeSurfer; i.e. in regions of high (extrinsic) curvature the vertex density is increased, leading to decreased face-wise surface areas. However, curvature is in itself highly non-normal, and computing a simple ratio of pial to white matter surface (Ratio_{pw}) would thus also result in a non-normal distribution. Histograms of the distribution of FSR and Ratio_{pw} illustrate that the non-linear term does indeed make the distribution more Gaussian-like (i.e. more normally distributed), while Ratio_{pw} is heavily skewed:

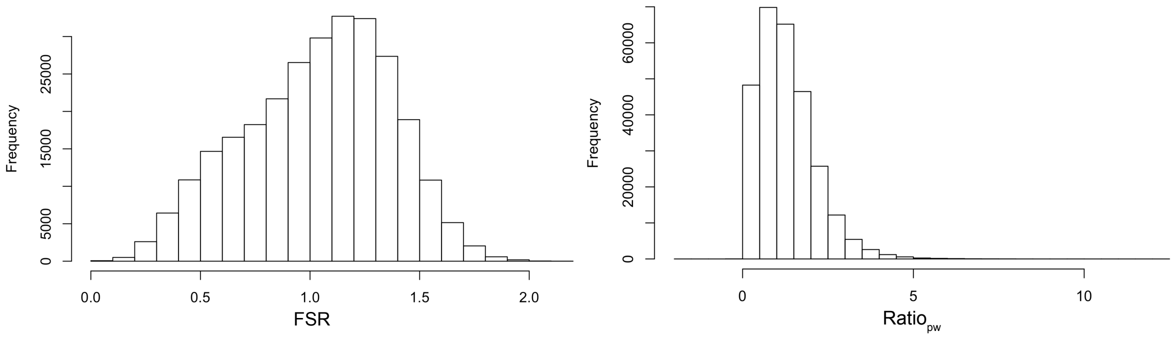

S3. Histograms depicting distributions of FSR and Ratio_{pw}. Histograms across all vertices for FSR and the ratio of pial to white surface area (Ratio_{pw}) for one example subject in native space.

For additional information, we computed Spearman's rank correlations between FSR and Ratio_{pw}, and mean curvature *H* for one representative participant on the vertex-level. We found that the correlations with *H* were almost identical for FSR (r = -0.8159) and Ratio_{pw} (r = -.08158). Thus, even when omitting the non-linear term in the computation of FSR, mean curvature *H* does not fully explain the surface ratio. This may be explained by the curvature of both pial and the white surfaces being involved in the ratio, but mean curvature *H* is based on the curvedness of only one surface (e.g. the white matter surface). Both FSR and Ratio_{pw}, however, capture information based on the geometries of two surfaces (both pial and white matter surfaces). In conclusion, although mean curvature *H* is highly correlated with measures of FSR, it cannot fully capture the neuroanatomical information provided by our feature.

S4. Computation of FSR.

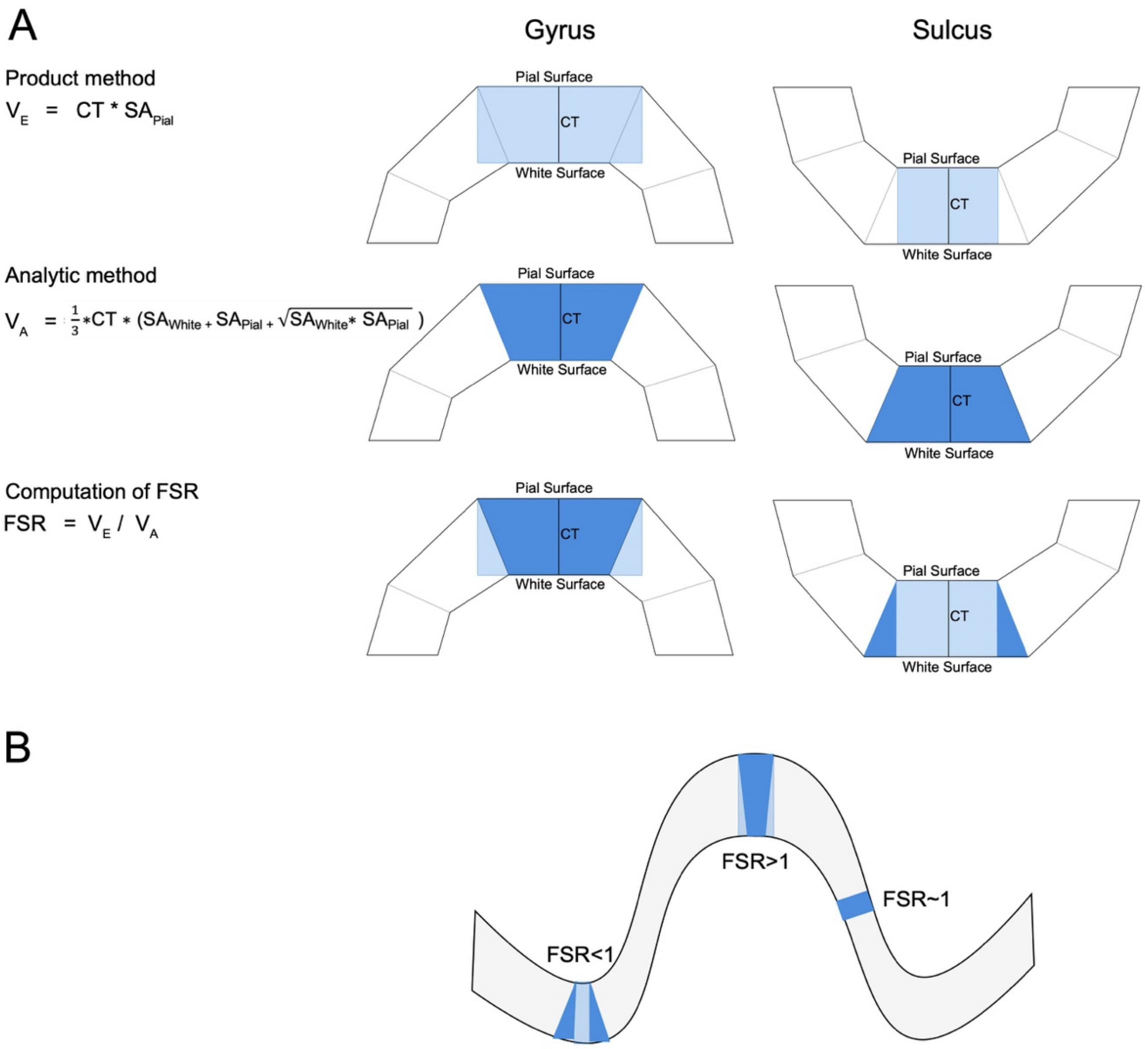

S4. Computation of FSR. (A) To capture volumetric gradients from the outer to the inner surface of the brain, the degree of FSR at each vertex was computed as a ratio between the estimated and the real volume at a given vertex. V_E is computed using the product method, which multiplies surface area and cortical thickness. This method overestimates cortical volume in gyri, and underestimates volume in sulci. V_A is computed using the bias-free analytic method, which computes the volume of a truncated pyramid without introducing errors. FSR is then computed as a ratio of V_E and V_A. (B) Overview of FSR values depending on the location of a vertex on the brain surface. FSR is smaller than 1 when the volume is *inward-weighted* (as in sulci), FSR is larger than 1 when the volume is *outward-weighted* (as in gyri), FSR is approximately 1 in areas between gyri and sulci, where the curvature of the brain is close to zero. *Note.* V_E, expected volume; V_A, analytical volume; CT, cortical thickness; SA_{pial}, surface area of pial surface; SA_{white}, surface area of white matter surface; FSR, Frustum Surface Ratio.

S5. Correlations between FSR and cortical descriptors.

A

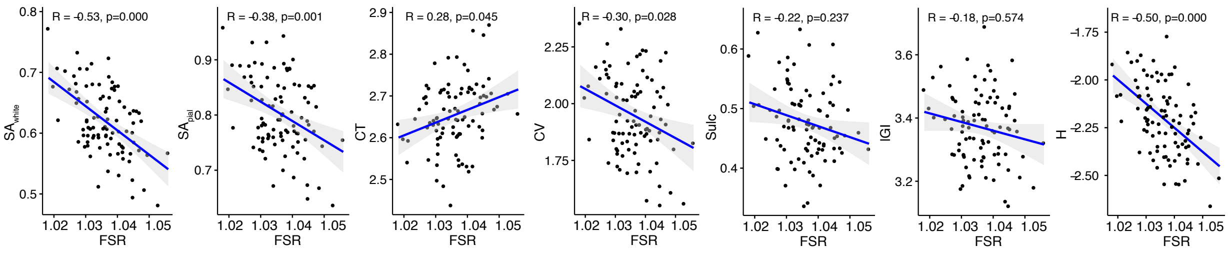

B

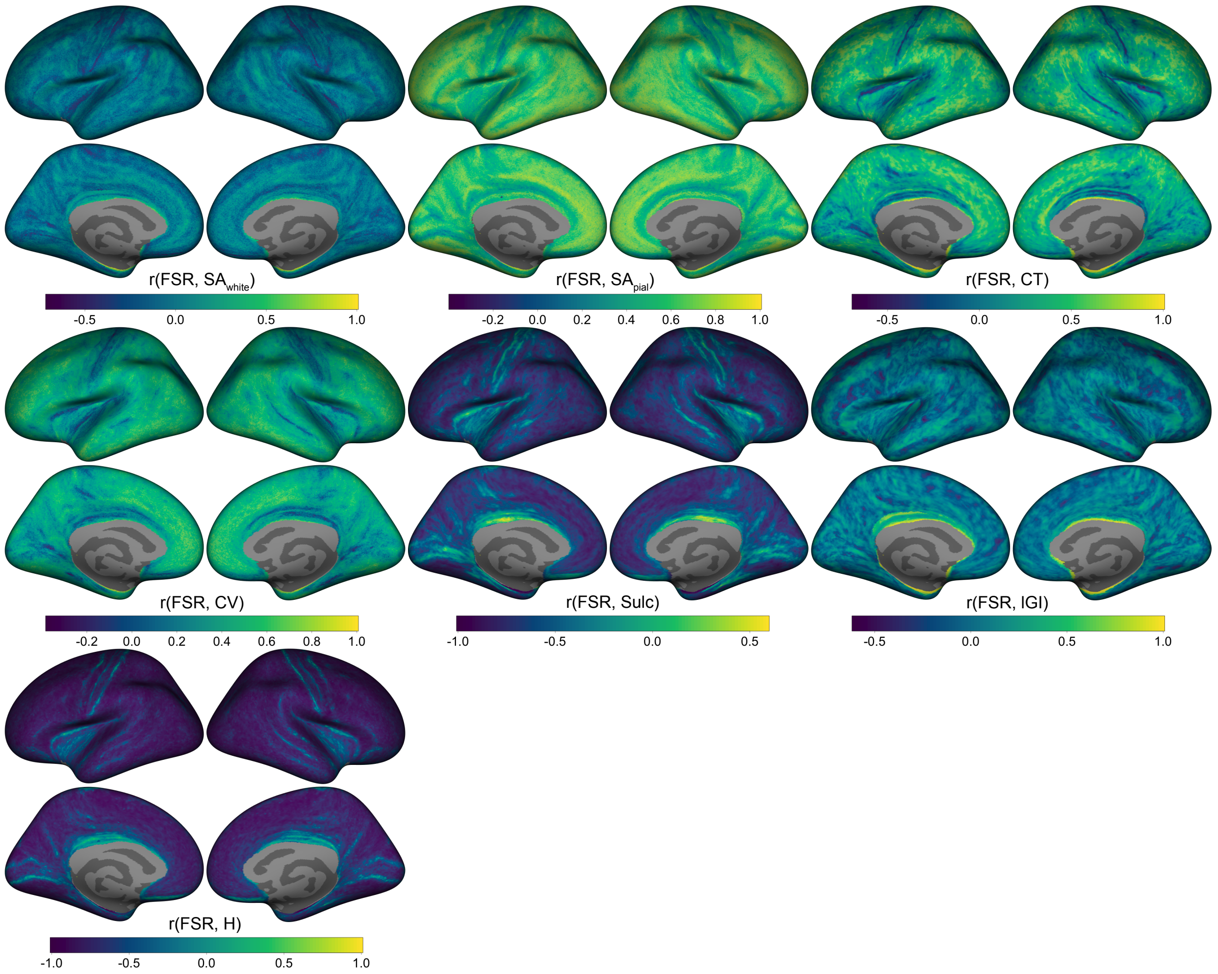

S5. Additional correlations between FSR and other cortical descriptors. (A) Correlations across subjects. Each point indicates the mean value per subject for the respective cortical descriptor. (B) Correlations across vertices. At each vertex, the correlation between descriptor pairs was computed for all typically developing controls (N=92). Negative correlations are displayed in blue to purple, positive correlations are displayed in green to yellow. Independent of the examined dimension, high correlations between FSR and mean curvature H are observed. This is consistent with the initial findings of H explaining the largest percentage of variability in FSR. *Note.* r, Spearman's Rank Correlation Coefficient; FSR, Frustum Surface Ratio; SA_{white}, Surface Area of white surface; SA_{pial}, Surface Area of pial surface; CT, Cortical Thickness; CV, Cortical Volume; Sulc, Sulcal Depth; lGI, local gyrification index; H, mean curvature.

S6. The effects of spatial smoothing on the distribution of FSR.

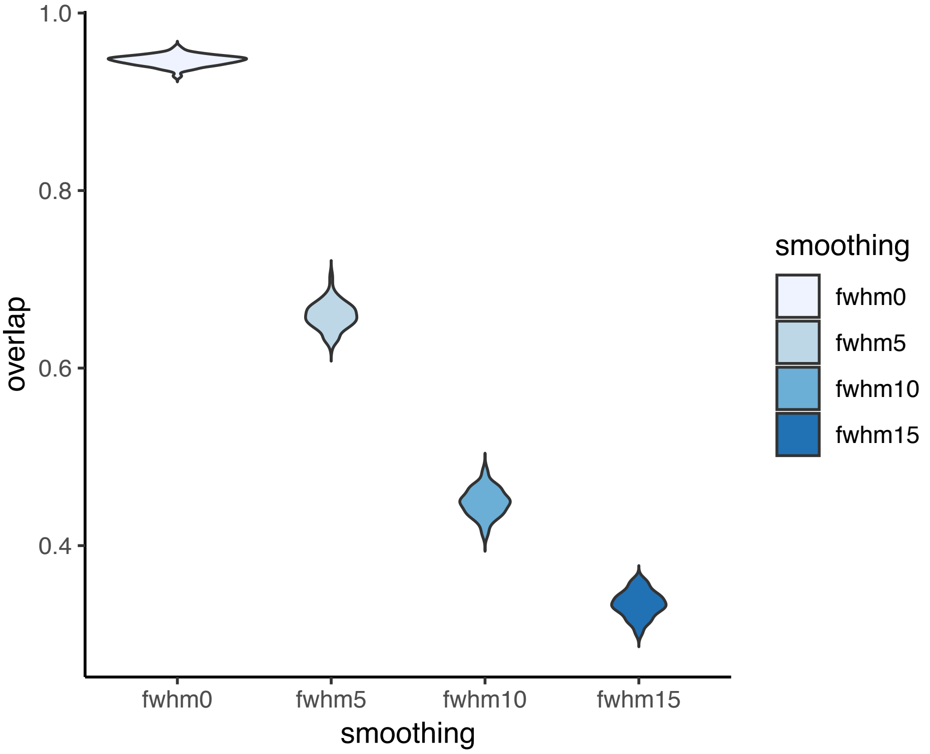

S6. Percentage overlap between measures of FSR in native space and several FWHM smoothing kernels. Across all neurotypical controls, the percentage overlap between FSR in native space and the respective smoothing kernels (i.e. FWHM0, FWHM5, FWHM10, and FWHM15) was computed. The resulting distributions are presented as violin plots depicting the respective median, minimum, and maximum. The degree of overlap was negatively correlated with width of smoothing kernel. *Note.* fwhm, full-width at half-maximum.

S7. Test of normality for measures of FSR.

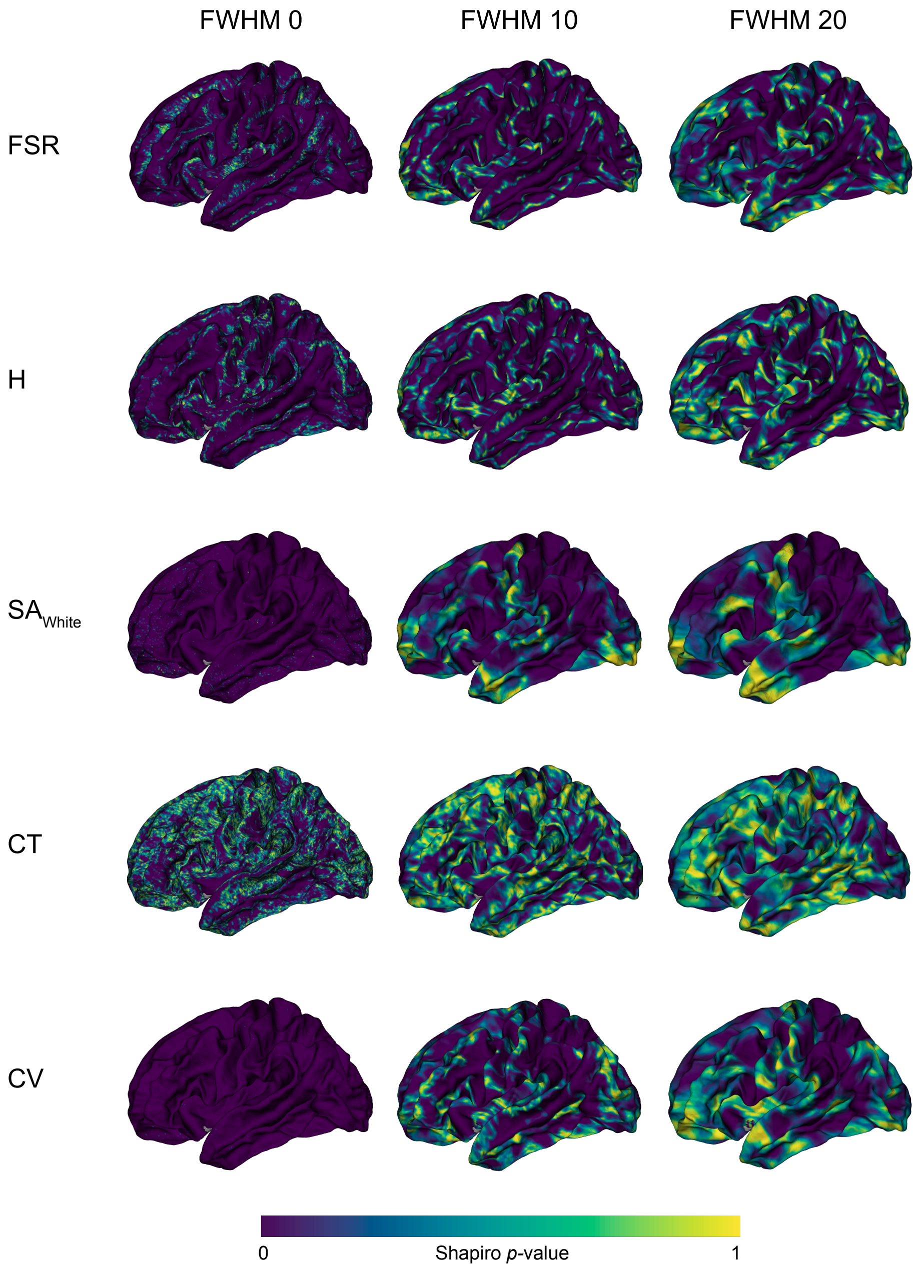

S7. Shapiro-Wilk test at each vertex for 92 neurotypical controls and five cortical measures. Displayed is the *p*-value as a measure for the probability of a normal distribution. For all features, the probability of the data being normally distributed without smoothing (FWHM 0) across participants was relatively low but increased with higher smoothing settings. *Note.* FWHM, full-width at half-maximum; FSR, Frustum Surface Ratio; H, mean curvature; SA_{white}, Surface Area of white surface; CT, Cortical Thickness; CV, Cortical Volume.

S8. Additional Group comparisons.

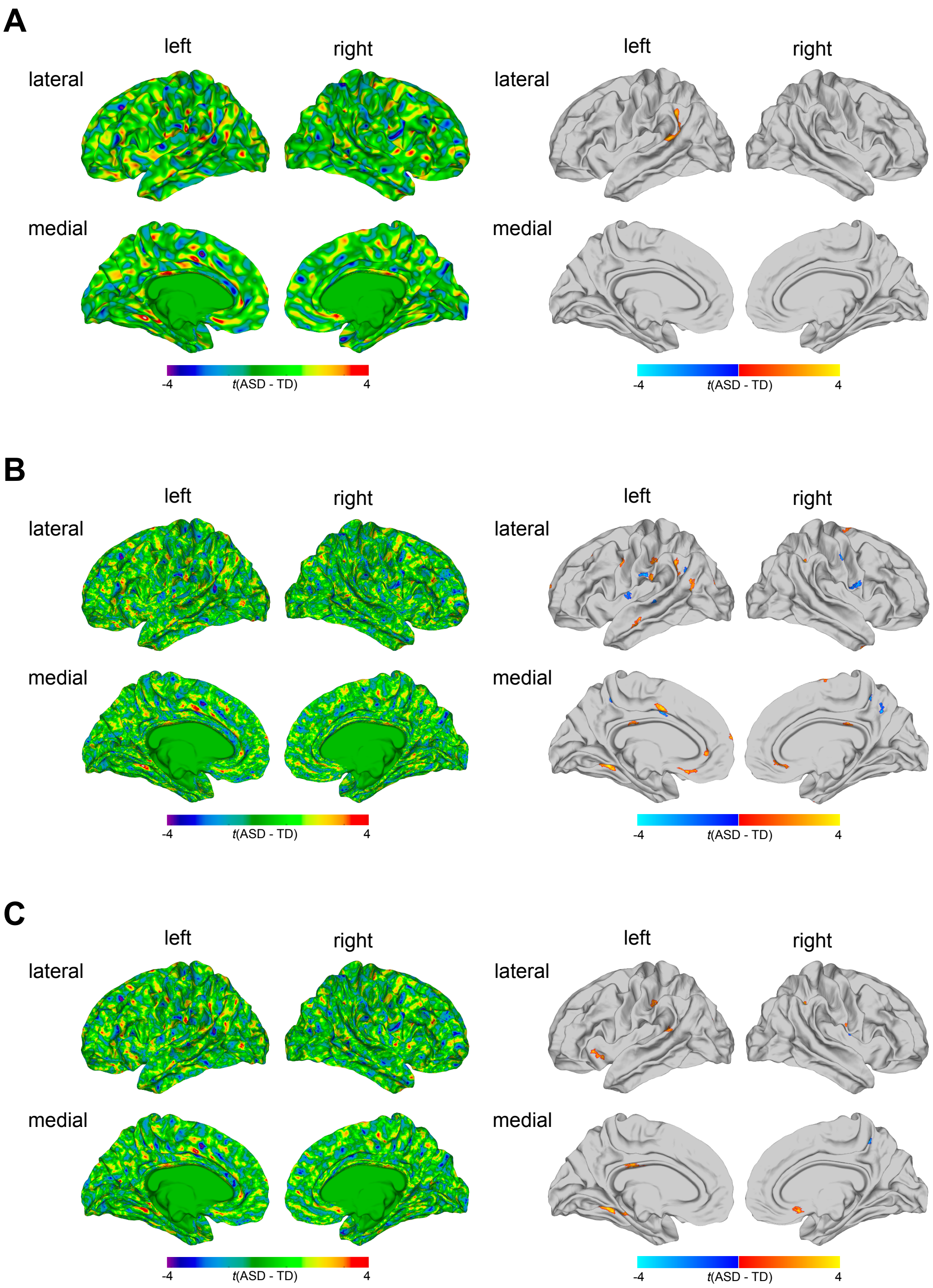

S8. Additional group comparisons of FSR between ASD and neurotypical controls. Between-group differences in FSR (A) with FSR smoothed at FWHM 5, (B) with FSR computed using FreeSurfer formula of analytical volume V_A, and (C) when including mean Euler number as covariate. Significant differences in FSR between individuals with ASD and TD while controlling for the effect of age, age-related interactions, sex, site, fsIQ (i.e. main effect of group). *Left panel*: unthresholded *t*-maps. *Right panel*: random field theory (RFT)-based cluster corrected *t*–maps ($p < 0.05$, 2-tailed). Here, significant increases in FSR are displayed in red to yellow, significant decreases are displayed in blue to cyan. *Note. t*, *t*-statistic; ASD, autism spectrum disorder; TD, typically developing controls.

S9. Analysis of Cortical Grey Matter Volume (CV).

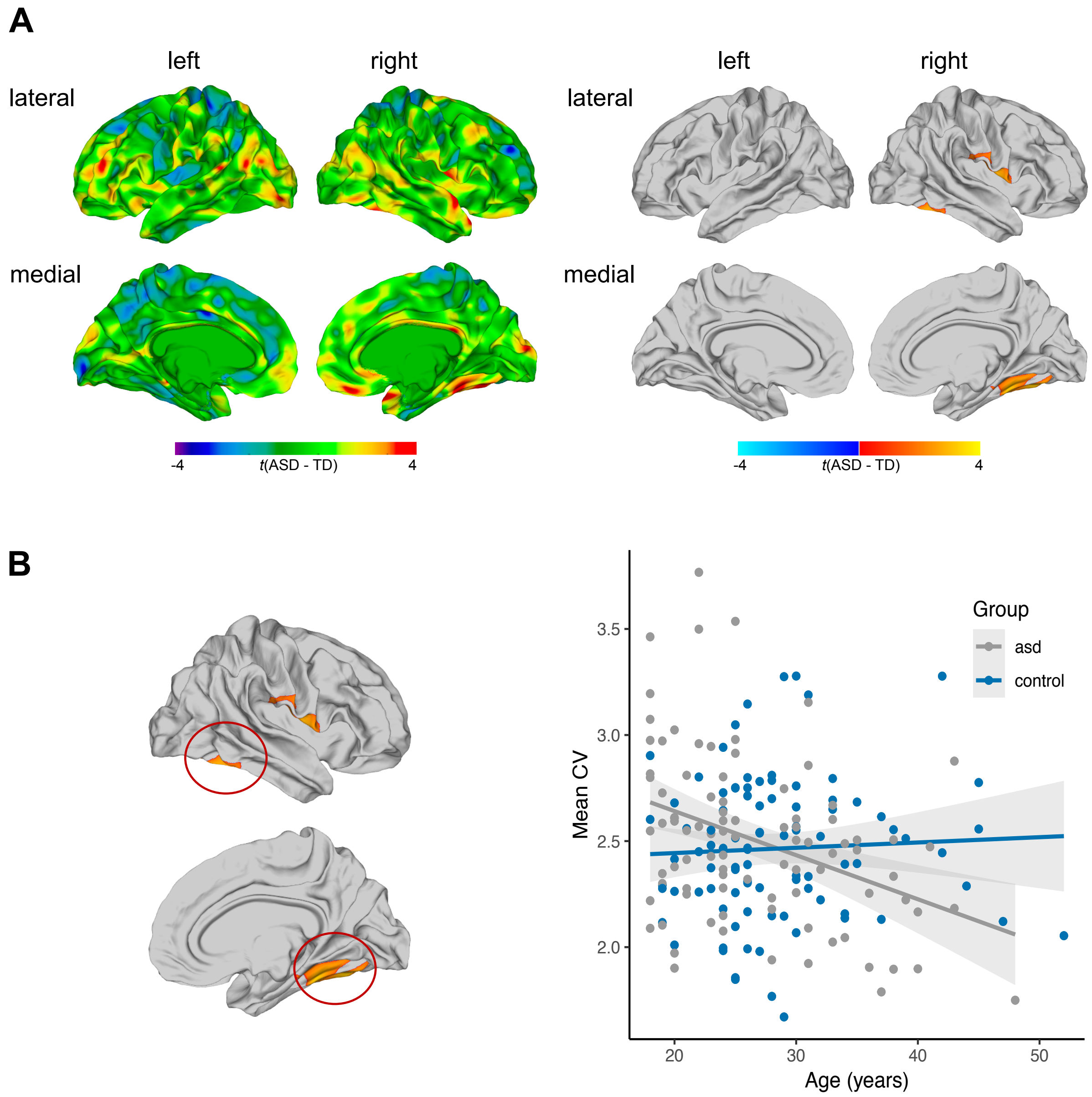

S9. Between-group differences and age-by-group interactions for CV. (A) Displayed are clusters with significantly increased CV in ASD compared to controls (red to yellow) while controlling for the effects of age, age-related interactions, gender, site, and fsIQ (i.e. main effect of group). There were no clusters with significantly decreased CV. *Left panel*: unthresholded *t*-maps. *Right panel:* random field theory (RFT)-based cluster corrected *t*–maps (*p*<0.05, 2-tailed). (B) Significant linear age-by-group interactions. *Left panel:* Significant linear age-by-group effects were observed in the right fusiform gyrus/inferior temporal cortex (RFT-based, cluster corrected, *p*<0.05). *Right panel:* scatter plot depicting age-related trajectories for individuals with ASD (grey) and neurotypical controls (blue). *Note. t*, *t*-statistic; ASD, autism spectrum disorder; TD, typically developing controls; CV, cortical grey matter volume.
